# First case of progressive solitary sclerosis with relapsing attacks: A 49‐year‐old woman

**DOI:** 10.1002/ccr3.9546

**Published:** 2024-10-31

**Authors:** Elnaz Asadollahzadeh, Vahid Shahmaei, Zahra Ebadi, Mohammad‐Sadegh Johari, Nasim Rezaeimanesh, Abdorreza Naser Moghadasi

**Affiliations:** ^1^ Multiple Sclerosis Research Center, Neuroscience Institute Tehran University of Medical Sciences Tehran Iran; ^2^ MAHAK Hematology Oncology Research Center (MAHAK‐HORC), MAHAK Hospital Shahid Beheshti University of Medical Sciences Tehran Iran; ^3^ Department of Radiology, Faculty of Medicine Aja University of Medical Sciences Tehran Iran

**Keywords:** methylprednisolone pulse therapy, relapsing attacks, rituximab, solitary sclerosis

## Abstract

This case highlights a rare form of solitary sclerosis with a relapsing pattern, contrasting with the typical single attack and nonrelapsing nature of the disease. Despite the lack of new lesions on MRI, This case demonstrates that, despite the rarity of this variant, a relapsing form of solitary sclerosis can occur and should be considered a differential diagnosis.

**Abstract:**

Progressive solitary sclerosis is characterized by isolated demyelinating damage to the central nervous system in the spinal cord and brainstem, leading to progressive motor impairment. We describe the case of a 49‐year‐old woman who suffered several recurrent attacks of right hemiparesis over time. The patient initially responded well to methylprednisolone pulse therapy without maintenance therapy. However, subsequent episodes resulted in mild residual symptoms and the progression of her condition. Clinical examination revealed normal cranial nerve function, decreased sensation in the right limbs, and abnormal signal findings on MRI of the cervical spine. Laboratory tests, vasculitis screening, cerebrospinal fluid (CSF) analysis, and brain/spinal cord angiography were all within normal limits. Based on these findings and the patient's clinical presentation, a diagnosis of progressive solitary sclerosis with relapsing attacks was made. Rituximab treatment was initiated with administration of a first dose of 1000 mg, followed by a second dose 6 months later.

## 1 INTRODUCTION

Progressive solitary sclerosis with relapsing attacks (PSSRA) is a rare form of demyelinating disease characterized by an isolated demyelinating lesion in the central nervous system (CNS), primarily affecting the spinal cord.^1^ Progressive solitary sclerosis is a demyelinating disease localized to a single site and characterized by progressive weakness primarily affecting the corticospinal tract.^2^ It is a recently recognized phenotype of multiple sclerosis (MS) that differs from the classic forms of MS and is characterized by the absence of relapses in most cases.^1^, ^3^ Although the specific cause of solitary sclerosis is unknown, it is believed to be an autoimmune disease in which the body's immune system attacks and damages the myelin sheath, the protective layer around nerve fibers in the CNS. The resulting destruction of the myelin sheath leads to impaired nerve signal transmission, leading to neurological deficits such as weakness, numbness, and sensory loss.^4^ We present the first reported case of PSSRA in a middle‐aged woman. This case represents a rare form of MS that deviates from the classic MS phenotypes and highlights the need for further research into the pathogenesis and optimal treatment of this unique disease presentation.

## 2 PRESENTATION

A 49‐year‐old woman presented with progressive right‐sided hemiparesis, initially beginning with an acute episode in 2007. She had no history or family history of neurological disorders and had no lifestyle factors such as smoking alcohol abuse or drug abuse. The patient was treated with methylprednisolone pulse therapy, which resulted in partial recovery, but mild residual symptoms persisted. Similar episodes occurred in 2012 and 2015, both of which also responded to methylprednisolone therapy, although residual symptoms persisted. The patient was briefly prescribed beta interferon as maintenance therapy, but this medication was discontinued. The patient's progressive right‐sided hemiparesis began with an acute attack in 2007 and over the past 2 years, the hemiparesis continued to worsen without any relapses or recovery periods. On neurological examination, cranial nerve function was found to be normal except for the Babinski sign on the right side. In addition, a limping gait was observed, suggesting motor impairment of the right extremity. The motor strength of the right upper and lower limbs was reduced to 4/5, while the left side remained normal.

## 3 DIAGNOSIS

**FIGURE 1:**
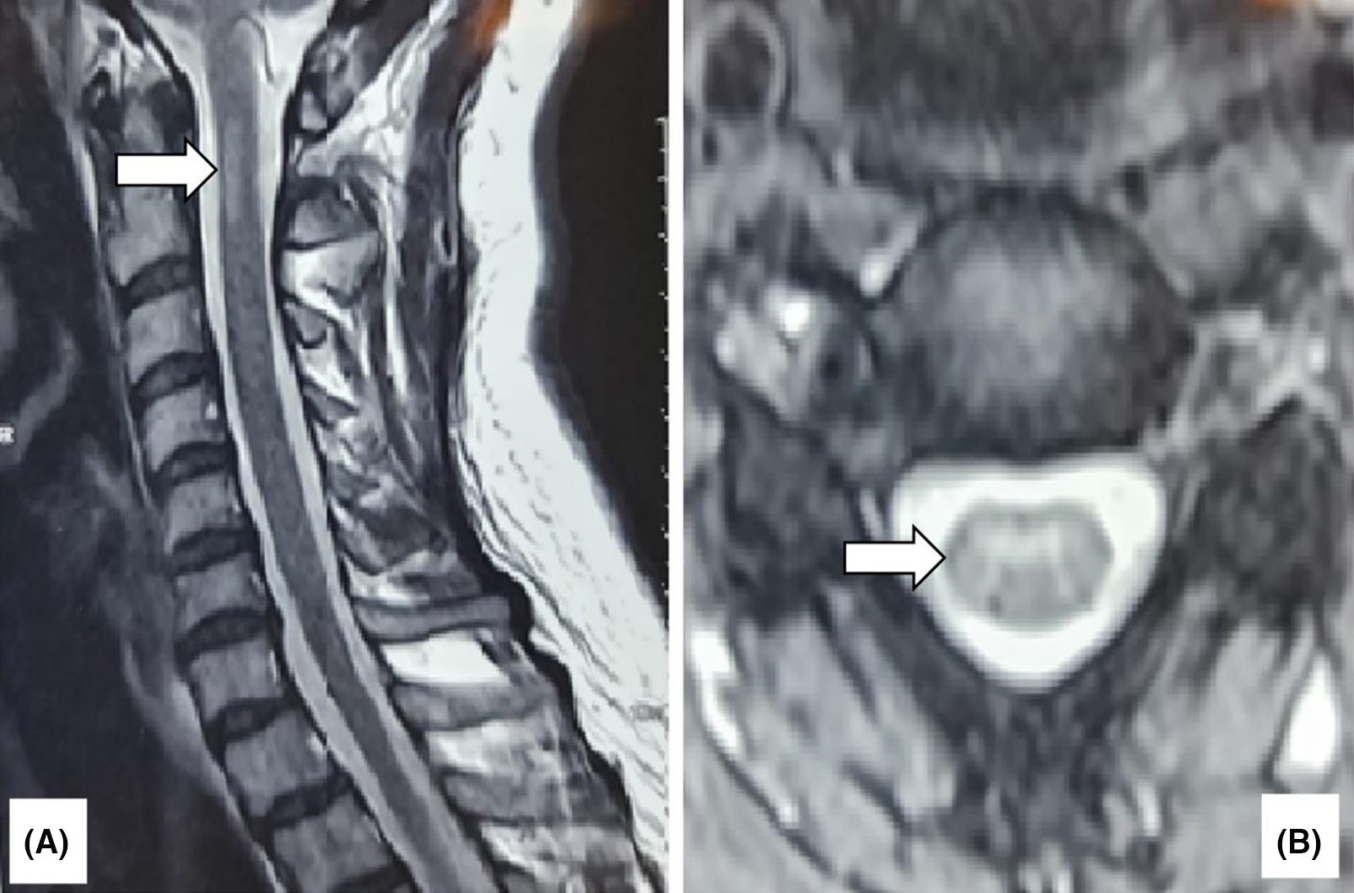
(A) Sagittal T2‐weighted turbo spin echo (TSE) image of the cervical cord. (B) Axial T2 gradient echo image of the cervical cord. A short segment hyper‐intense lesion is observed in the anterior central cord at the C2 level. There is no evidence of cord expansion.

The patient's brain MRI showed no signal changes in the 3D T2 FLAIR sequence. However, an MRI of the cervical spine (Figure 1) showed an area of increased signal intensity in the anterocentral region of C1‐C2 without contrast enhancement. This anomaly showed no changes in size, location, or signal intensity from the initial attack in 2007. CSF analysis was within normal limits. The cell count was less than 5 cells per microliter and the protein content of 45 milligrams per deciliter is within the normal range for CSF. Oligoclonal bands (OCB) were not detected in the CSF and the immunoglobulin G index (IgG) was normal. To exclude neuromyelitis optica spectrum disorder (NMOSD) as a possible diagnosis, an aquaporin‐4 Ab was performed, and the results were negative. To rule out MOG‐IgG‐associated disease (MOG‐AD), a test for MOG (myelin oligodendrocyte glycoprotein) was conducted, yielding a negative result. Biochemical tests including blood count, liver function tests, kidney function tests, thyroid function tests and vitamin and mineral levels, as well as vasculitis screening (erythrocyte sedimentation rate (ESR), C‐reactive protein (CRP), antinuclear antibodies (ANA)), lupus anticoagulant (LA), anti‐cardiolipin antibodies (ACL Ab), SSA, SSB and antineutrophil cytoplasmic antibodies (ANCAs), showed no abnormalities.

The patient's vitamin B12 level was within the normal range, meaning that a vitamin B12 deficiency could be ruled out as a possible cause of her symptoms. The evaluation of an underlying cancer involves a variety of diagnostic tests and procedures. Blood tests, including a complete blood count (CBC), metabolic panel, and tumor markers. Imaging techniques such as CT scans and FDG‐PET scans gave negative results and excluded paraneoplastic myelitis as a contributing factor to the patient's condition. Thoracic MRI and digital subtraction angiography (DSA) results of the brain/spinal cord were also normal and excluded the presence of vascular malformations. Based on the patient's clinical presentation, imaging findings, and exclusion of other differential diagnoses, a diagnosis of PSSRA was made.

## 4 TREATMENT

Given the progressive nature of the disease, with recurrent episodes of right hemiparesis that were positively responsive to methylprednisolone but accompanied by mild residual symptoms, treatment with Rituximab was initiated to target B‐cell involvement, by administering a first dose of 1000 mg, followed by a second dose 6 months later.

The patient was followed up for 12 weeks after receiving the treatment. During this follow‐up period, the patient's neurological examination revealed improved motor strength on the right side, with the patient's Medical Research Council (MRC) score increasing from 4/5 to 4+/5.

## 5 DISCUSSION

This case report established the diagnosis of “PSSRA” based on clinical presentation, imaging findings, and elimination of other likely diagnoses, providing insight into this rare variant of demyelinating disease. In this study, the patient presented motor impairment predominantly on the right side, as evidenced by a Babinski sign and decreased motor strength in the right upper and lower limbs, while the left side remained normal.

Progressive solitary sclerosis is a rare form of demyelinating disease characterized by an isolated CNS demyelinating lesion in the spinal cord. It is a unifocal demyelinating disease of the corticospinal tract with downstream progressive weakness. Most patients with MS have a relapsing–remitting course characterized by subacute, inflammatory clinical attacks that usually resolve spontaneously or with treatment. To date, all cases of solitary sclerosis reported in the literature have occurred in a monophasic form. However, our case reports show a relapsing form that resembles MS. One possible theory is that inflammatory processes occur at the microscopic level and may lead to relapse of clinical symptoms without visible changes on conventional MRI. These processes may involve subtle changes in the blood–brain barrier or glial cell activation, which may contribute to the recurrence of symptoms without significantly altering the macroscopic appearance of the lesion. Further research is needed to elucidate the precise mechanisms underlying symptom relapse in patients with solitary sclerosis with stable MRI findings. In addition, this point emphasizes on the usage of advanced MRI for better understanding the nature of this disease. Advance imaging may show radiological changes.

Similar to our case, Tavee, Jinny O. et al.^5^ reported a 25‐year‐old woman with a solitary brain lesion who presented with spastic hemiparesis. The patient showed increasing weakness and spasticity over the years, which resembled our case. In rare cases, it has been documented that solitary sclerosis may be associated with a gradual decline in motor function over a prolonged period, representing a distinct clinical course.^6^ The CSF analysis for our patient yielded normal results, which aligns with the observations in a case study by Barakat, Benan, et al.^7^ The case study described a patient with progressive solitary sclerosis, and in that instance, the CSF analysis indicated a white blood cell count of 1 and no significant abnormalities. In another study, CSF samples from patients with progressive motor impairment secondary to severe demyelinating lesions of the CNS, including progressive solitary sclerosis, were examined. The results of CSF analysis were unremarkable in these patients.^6^ The results of CSF analyses in the studies mentioned were normal, meaning that there were no significant abnormalities or markers related to the neurological diseases examined, including progressive solitary sclerosis.

The MRI of the brain showed no significant abnormalities, while the MRI of the cervical spine showed unusually high signal intensity in the C1‐C2 region without enhancement, and the MRI images of this region showed no significant changes in the intensity and position of the signal from one scan to the next showed. in line with the first attack. While brain MRI showed no significant abnormalities, cervical spine MRI showed a stable abnormality in the upper cervical spinal cord. Individual MRI lesions were consistently located in characteristic regions associated with demyelinating MS plaques, particularly along corticospinal tracts, including the lateral columns of the spinal cord, ventral horns, and brainstem.^8^ In our study, we encountered a patient who presented with a single demyelinating lesion and progressive neurological symptoms, similar to the cases reported in the research of Lee, Lisa Eunyoung et al. have been described.^9^ In this study, the authors examined the advanced MRI findings in two cases of progressive solitary sclerosis and reported a lower N‐acetyl aspartate (NAA)/total creatine (tCr) ratio compared to patients with MS and healthy controls. Although we did not perform a similar quantitative MRI analysis in our case, the results of Lee, Lisa Eunyoung et al. suggest that patients with progressive solitary sclerosis may have metabolic abnormalities reflected, for example, in a reduced NAA/tCr ratio.

Research on the use of Rituximab in the treatment of progressive solitary sclerosis is limited. However, there have been studies examining its effectiveness in reducing disease activity in relapsing–remitting MS.^10^ In a case report of a patient with progressive solitary sclerosis who suffered from diplopia, corticosteroid pulse therapy, and rituximab did not produce significant improvement. However, partial improvement was observed when cyclophosphamide was added to the treatment regimen.^11^ Further research is needed to determine the effectiveness of rituximab specifically in progressive solitary sclerosis.

## 6 CONCLUSION

This case demonstrates a rare form of solitary sclerosis with a relapsing pattern, in contrast to the typical single attack and nonrelapsing nature of the disease. Although no new lesions are seen on MRI, this case demonstrates that, despite the rarity of this variant, a recurrent form of solitary sclerosis can occur and should be considered a differential diagnosis. This could be described as a “solitary demyelinating lesion with an atypical, progressive‐recurrent course.”

## AUTHOR CONTRIBUTIONS

**Elnaz Asadollahzadeh:** Conceptualization; investigation; project administration; supervision; writing – original draft. **Vahid Shahmaei:** Conceptualization; writing – original draft; writing – review and editing. **Zahra Ebadi:** Data curation; validation; writing – review and editing. **Mohammad‐Sadegh Johari:** Conceptualization; investigation; writing – original draft; writing – review and editing. **Nasim Rezaeimanesh:** Writing – original draft; writing – review and editing. **Abdorreza Naser Moghadasi:** Conceptualization; data curation; supervision; writing – original draft; writing – review and editing.

## FUNDING INFORMATION

This research did not receive any specific grant from funding agencies in the public, commercial, or not‐for‐profit sectors.

## CONFLICT OF INTEREST STATEMENT

The authors declare that there is no conflict of interest.

## ETHICS STATEMENT

N/A.

## CONSENT

The objectives of the study were explained to the patient and written informed consent has been obtained from the patient.

## Data Availability

The data is available upon a reasonable request.
